# Supplementary material for: Sun-Protective Clothing Worn Regularly during Early Childhood Reduces the Number of New Melanocytic Nevi: The North Queensland Sun-Safe Clothing Cluster Randomized Controlled Trial
Source: Cancers (Basel). 2023 Mar 14;15(6):1762. doi: 10.3390/cancers15061762 (PMC10046807; doi:10.3390/cancers15061762)
Supplement: Supplementary file 1 [file cancers-15-01762-s001.zip › Suppl Table S1 130323.pdf]

Table S1. Summary of methods used in cluster-randomized childcare centers (clusters) and participating children in Townsville, Queensland, Australia, between November 1999 and July 2005.

<table>
<tr><th colspan="2">Both Groups</th></tr>
<tr><td colspan="2">• Shade audit conducted at childcare centers. We determined the ratio of covered to uncovered play areas, measured penetration of UVR vertically and horizontally for 5+ specific shade structures at each center.
• Subjects were recruited from intervention and control childcare centers (Nov 1999-July 2002) by sending parents/carer an information sheet, consent form and baseline questionnaire.
• Baseline phenotypic assessment conducted (hair, eye and skin-color assessed, skin reflectance measured for sun-protected inner-upper arm and dorsum of hand), weight and height measured, and full-body skin examination (excluding buttocks, genitals and scalp) performed to record melanocytic nevi (MN) by size (<2mm, ≥2mm, ≥3mm, ≥4mm, ≥5mm) and anatomical body-site (total 30 body-sites); freckling assessed for face, arms and shoulders.
• Skin examination for MN (30 body-sites) repeated annually for up to 4 years (end-count of MN performed where possible before eligible child left center) together with phenotypic assessment (as described above). Parents/carer sent an annual summary of the number of MN their child has.
• Questionnaire completed by a senior staff member in each unit (age group) in each participating childcare center showing time and duration of outdoor activities and use of various forms of sun protection used.
• Regular sun-exposure questionnaires sent to parents/carer(s) of participating children.</td></tr>
<tr><th>Control-arm</th><th>Intervention-arm</th></tr>
<tr><td>• Observations of sun-protective behaviors undertaken at childcare centers when obtaining lists of potential participants (during recruitment phase Nov 1999-June 2002) and when conducting baseline/annual MN examinations or end counts.
• No study-garments distributed at these centers or to these children. Staff continued to provide usual care in accordance with the center's sun-protection policy.</td><td>• Observations of compliance (% children wearing hat and study garments) undertaken twice/week when collecting laundry as well as when conducting baseline/annual MN examinations or end counts.
• Intervention childcare centers were provided with enough study-garments to clothe all enrolled children. This included (a) crew-neck T-shirts made from 180gm 100% cotton rated as offering"very good" UVR protection [54] in sizes 0, 1, 2, 3, 4, 6, & 8, with sleeves 20.7% longer than Australian standard sizing to reach to below the elbows (Mean UPF for new shirts ranged from 32.1-44.9, increasing to 91.4-151.2 after 3.5 months of repeated use & laundering); (b) UPF 40 nylon taslon knee-length shorts in sizes 1, 2, 3, 4, 6 & 8; (c) long-sleeve UPF 40 taslon shirts with crew neck in sizes 1, 2, 4, 6, 8.
• Clothing maintained by laundry service. Monitoring of weekly laundry volumes allowed prompt investigation of lower-than usual clothing usage.
• Staff instructed on correct use of study-garments (must cover arms to below the elbows & legs to below knees.
• Laminated signs put on exit doors to remind staff to dress children before going outside.
• Parents educated about sun-protection & correct use of garments at the center & at home (education session & instruction sheet).
• Legionnaire hats and protective swimwear provided for home-use just before summer each year: Long-sleeved UPF 40 nylon taslon shirt first summer & commercially available UPF 50+ Lycra (nylon elastane) cover-up suit with long sleeves & full/knee-length legs thereafter.</td></tr>
</table>

Abbreviations: MN Melanocytic nevus (singular), nevus (plural); UVR Ultraviolet Radiation.
