# Supplementary material for: Sun-Protective Clothing Worn Regularly during Early Childhood Reduces the Number of New Melanocytic Nevi: The North Queensland Sun-Safe Clothing Cluster Randomized Controlled Trial
Source: Cancers (Basel). 2023 Mar 14;15(6):1762. doi: 10.3390/cancers15061762 (PMC10046807; doi:10.3390/cancers15061762)
Supplement: Supplementary file 1 [file cancers-15-01762-s001.zip › Suppl Table S2 CONSORT Checklist Extension for Cluster Trials 2012 Checklist Cancers 13March23.pdf]

Table S2. CONSORT 2010 checklist including extension for cluster designs [57].

Section/Topic	Item No	Standard Checklist item	Extension for cluster designs	Page No *
**Title and abstract**				
	1a	Identification as a randomised trial in the title	Identification as a cluster randomised trial in the title	**p1**
	1b	Structured summary of trial design, methods, results, and conclusions (for specific guidance see CONSORT for abstracts)	See table 2	**p1**
**Introduction**				
**Background and objectives**	2a	Scientific background and explanation of rationale	Rationale for using a cluster design	**P3-4 P7**
	2b	Specific objectives or hypotheses	Whether objectives pertain to the cluster level, the individual participant level or both	**p1, p3-4**
**Methods**				
**Trial design**	3a	Description of trial design (such as parallel, factorial) including allocation ratio	Definition of cluster and description of how the design features apply to the clusters	**P4-9, Supplementary Table S1**
	3b	Important changes to methods after trial commencement (such as eligibility criteria), with reasons		**N/A**
**Participants**	4a	Eligibility criteria for participants	Eligibility criteria for clusters	**P4 Methods** (inclusion criteria clusters) **p7** (inclusion criteria children)
	4b	Settings and locations where the data were collected		**P4**
**Interventions**	5	The interventions for each group with sufficient details to allow replication, including how and when they were actually administered	Whether interventions pertain to the cluster level, the individual participant level or both	**P4-7, Table S1** (Interventions pertain to cluster level)

**Outcomes**	6a	Completely defined pre-specified primary and secondary outcome measures, including how and when they were assessed	Whether outcome measures pertain to the cluster level, the individual participant level or both	**P7**<br><br>cluster level
	6b	Any changes to trial outcomes after the trial commenced, with reasons		**N/A**
**Sample size**	7a	How sample size was determined	Method of calculation, number of clusters(s) (and whether equal or unequal cluster sizes are assumed), cluster size, a coefficient of intracluster correlation (ICC or *k*), and an indication of its uncertainty	**P13**
	7b	When applicable, explanation of any interim analyses and stopping guidelines		**N/A**
**Randomisation:**				
**Sequence generation**	8a	Method used to generate the random allocation sequence		**P4**
	8b	Type of randomisation; details of any restriction (such as blocking and block size)	Details of stratification or matching if used	Matched on shade and SES<br><br>**P4**
**Allocation concealment mechanism**	9	Mechanism used to implement the random allocation sequence (such as sequentially numbered containers), describing any steps taken to conceal the sequence until interventions were assigned	Specification that allocation was based on clusters rather than individuals and whether allocation concealment (if any) was at the cluster level, the individual participant level or both	**P4**(allocation based on clusters)<br><br>**P6** (allocation concealment not possible – reasons explained in text)
**Implementation**	10	Who generated the random allocation sequence, who enrolled participants, and who assigned participants to interventions	Replace by 10a, 10b and 10c	**See 10a, 10b, 10c (below)**
	10a		Who generated the random allocation sequence, who	**P4** (PGB random allocation & assigned clusters to intervention;

			enrolled clusters, and who assigned clusters to interventions	SLH enrolled clustered)
	10b		Mechanism by which individual participants were included in clusters for the purposes of the trial (such as complete enumeration, random sampling)	**P4** Allocation was based on clusters rather than individuals.
	10c		From whom consent was sought (representatives of the cluster, or individual cluster members, or both), and whether consent was sought before or after randomisation	**P4** (centers) **& P6** (parents of eligible children)<br><br>Both
**Blinding**	11a	If done, who was blinded after assignment to interventions (for example, participants, care providers, those assessing outcomes) and how		**N/A reasons explained on p6**
	11b	If relevant, description of the similarity of interventions		**N/A**
**Statistical methods**	12a	Statistical methods used to compare groups for primary and secondary outcomes	How clustering was taken into account	**P8-9**
	12b	Methods for additional analyses, such as subgroup analyses and adjusted analyses		**P8-9**
**Results**				
**Participant flow (a diagram is strongly recommended)**	13a	For each group, the numbers of participants who were randomly assigned, received intended treatment, and were analysed for the primary outcome	For each group, the numbers of clusters that were randomly assigned, received intended treatment, and were analysed for the primary outcome	**P5**
	13b	For each group, losses and exclusions after randomisation, together with reasons	For each group, losses and exclusions for both clusters and individual cluster members	**P5 & p8 & p10**

**Recruitment**	14a	Dates defining the periods of recruitment and follow-up		**P5, p12**
	14b	Why the trial ended or was stopped		**P7** (concluded July 2005)
**Baseline data**	15	A table showing baseline demographic and clinical characteristics for each group	Baseline characteristics for the individual and cluster levels as applicable for each group	**Table 1** (clusters) **Table 2** (children)
**Numbers analysed**	16	For each group, number of participants (denominator) included in each analysis and whether the analysis was by original assigned groups	For each group, number of clusters included in each analysis	**P9 &P10**
**Outcomes and estimation**	17a	For each primary and secondary outcome, results for each group, and the estimated effect size and its precision (such as 95% confidence interval)	Results at the individual or cluster level as applicable and a coefficient of intracluster correlation (ICC or k) for each primary outcome	**Table 3 p12-13** (95%-Ci for median diff provided)
	17b	For binary outcomes, presentation of both absolute and relative effect sizes is recommended		**N/A**
**Ancillary analyses**	18	Results of any other analyses performed, including subgroup analyses and adjusted analyses, distinguishing pre-specified from exploratory		**p12** (baseline characteristics of lost to follow-up children compared to 544 children with at least 1st follow-up exam)
**Harms**	19	All important harms or unintended effects in each group (for specific guidance see CONSORT for harms[1])		**P10**
**Discussion**				
**Limitations**	20	Trial limitations, addressing sources of potential bias, imprecision, and, if relevant, multiplicity of analyses		**P20**
**Generalisability**	21	Generalisability (external validity, applicability) of the trial findings	Generalisability to clusters and/or individual participants (as relevant)	**P20**

**Interpretation**	22	Interpretation consistent with results, balancing benefits and harms, and considering other relevant evidence	**p1, 20-21**
**Other information**			
**Registration**	23	Registration number and name of trial registry	**p1 & p4**
**Protocol**	24	Where the full trial protocol can be accessed, if available	**P4**
**Funding**	25	Sources of funding and other support (such as supply of drugs), role of funders	**P21**

** Note: page numbers optional depending on journal requirements*
